# Gender Differences Related to Spirituality, Coping Skills and Risk Factors of Suicide Attempt: A Cross-Sectional Study of French Adolescent Inpatients

**DOI:** 10.3389/fpsyt.2021.537383

**Published:** 2021-06-25

**Authors:** Bojan Mirkovic, Vincent Belloncle, Hugues Pellerin, Jean-Marc Guilé, Priscille Gérardin

**Affiliations:** ^1^Department of Child and Adolescent Psychiatry, Nouvel Hopital de Navarre, Normandie Université, Evreux, France; ^2^Équipe INSERM ≪ Psychiatrie du Développement ≫, Centre de Recherche en Épidémiologie et Santé des Populations, UMR 1018, Université Paris-Saclay - UVSQ, Versailles, France; ^3^Department of Child and Adolescent Psychiatry, CHU Rouen/CH-Le Rouvray, Normandie Université, Rouen, France; ^4^Department of Child and Adolescent Psychiatry, Pitié-Salpêtrière AP-HP, UPMC, Sorbonne Université, Paris, France; ^5^Department of Child and Adolescent Psychiatry, University Hospital of Amiens, Université Jules Verne, Amiens, France; ^6^Laboratoire CRFDP, Normandie Université, Rouen, France

**Keywords:** adolescent, attempt suicide, coping, gender, spirituality

## Abstract

**Background:** Suicide attempts in adolescence represent a major public health concern, since these behaviors are associated with psychosocial burden and an increased risk of suicide. This cross-sectional study aimed to explore possible gender differences related to protective and risk factors in adolescents who have attempted suicide.

**Methods:** Participants were French adolescents hospitalized for attempt suicide in five French pediatric departments. The participants were evaluated on 12 instruments measuring individual risk and protective factors.

**Results:** Our sample included 320 adolescents aged 13–17 years (M = 14.43, SD = 1.29), of whom 82% were female and 35% were repeat attempters. Boys had greater difficulties at school and used more lethal means such as strangulation. We failed to find any differences between the two groups as regards the main Axis I psychiatric diagnoses. Boys tend to use more non-productive coping skills such as *tension reduction* or *wishful thinking* and girls use more reference to other strategies such as *seeking social support*. Although spirituality scores were low overall sample, they were significantly higher among girls.

**Conclusions:** In the end, we find little difference between the two groups in terms of risk factors. However, we have shown gender differences in spirituality and some coping strategies. These results should be taken into consideration when designing suicide prevention programs.

## Introduction

Suicide is the second leading cause of death among adolescents and young adults and represents a major public health concern (1). Epidemiological studies of adolescent suicide report higher rates of non-fatal suicidal behavior in women, suggesting that there is a gender difference in suicidal behavior during adolescence (2–6). In general, suicidal thoughts and suicide attempts (SA) are more common in women than in men, but the suicide death rate is higher in men. This characteristic is known as the “gender paradox” in suicide (7, 8). It is recognized that social factors play an important role in the differences in mental health between the sexes (9, 10). Several hypotheses have been proposed to explain this difference, such as the diversity of gender-based cultural norms (7) or the greater exposure of women to negative psychosocial experiences. In line with this, the recent study by Chang et al. (11) based on World Health Organization data showed that gender inequality (measured by the Gender Inequality Index) contributed to the overall variations in suicide in the world. Similarly, Sigurdson et al. (12) showed in a longitudinal study that women who were victims of bullying showed a decrease in suicidal thoughts between adolescence and adulthood, while men who were victims of bullying showed an increase in SA during the same period. Indeed, it is important to remember that exposure to childhood abuse and neglect has been associated with increased depression and suicidal behavior (13, 14).

Another explanation for the gender paradox is that adolescent boys are more likely than adolescent girls to choose more lethal suicide methods such as hanging or firearms (15). The choice of method may be related to the intention to commit suicide. The Europe-wide study by Freeman et al. (16) showed a significant association between suicidal intention and gender, with “serious suicide attempts” being assessed as significantly more common in men than in women.

On a neurobiological level, some recent studies have shown gender differences for glutaminergic activity (17) physical activity (18), sensory processing (19, 20) or sleep time (21).

It has been reported that the direct effect of lack of sleep on increased suicidal ideation is greater in adolescent girls than in adolescent boys (21). Lastly, differences were observed in the use of health care services. The study by Gontijo Guerra et al. (22) found that girls were more likely than boys to have used health services in the year before they died by suicide. Given the reality of such gender differences, the accepted approaches to suicide prevention should also be expected to take gender differences into account. Unfortunately, gender differences in terms of risk and protective factors for SA in adolescent have received insufficient attention. In this cross-sectional study, we propose to partially fill this gap.

Several studies have evaluated gender differences in mental disorders and suicidality. The Europe-wide study by Boyd et al. (23) shows in a consistently way that women have more internalized disorders (for example, mood disorders) while men have more externalized disorders (for example, behavioral disorders). The higher rates of internalized disorders among women could result in higher rates of SA, while the higher rates of externalized behavior among men could lead to a higher death rate by suicide (9, 24–26). A recent meta-analysis by Miranda-Mendizabal et al. (26) identifies eating disorders, post-traumatic stress disorder, bipolar disorder and depressive disorders as being risk factors for SA which are specific to women. However, the authors also report that previous mental or substance-abuse disorders and exposure to interpersonal violence were common risk factors for both sexes. Protective factors involved in moderating suicidal risk during adolescence include cognitive characteristics such as productive coping strategies, spirituality or religiosity, reasons for living, social and family support, doing well at school or regular physical activity (27–30). We selected productive coping skills and spirituality because these individual factors have been identified as being proximal to suicidal behavior, modifiable, and measurable (31). Regarding coping skills, a distinction is usually made between two coping styles: functional (productive) and dysfunctional (non-productive) (32). Non-functional coping strategies appear to be associated with depressive symptoms and suicidal ideation in adolescent clinical (29, 33) and community populations (28). Little data is available regarding gender differences because most studies have failed to take gender into consideration. It has been suggested that women were more likely than men to use emotion-based rather than problem-based coping strategies (33, 34). Spiritual and religious behaviors are difficult to define and are subject to controversy. Spirituality refers to an individualistic, open, liberating and ultimately subjective quest, while religiosity requires a somewhat narrower characterization (35). Research on the impact of spirituality on adolescent developmental outcomes suggests that high levels of spirituality are associated with a better overall development (36). Spirituality seems to be a moderator for depression, hopelessness and adolescent SB (37). Rasic et al. (38) showed that religion had a protective effect for depression and suicide ideation in adolescent populations. However, few studies have examined gender differences in clinical populations. Breton et al. (39) showed that self-discovery from the Spirituality Scale is a significant protective factor for both girls and boys in the community but reached the significance level only for boys in the adolescent clinical population.

The main objective of this descriptive study was to examine gender differences as regards the main risk and protective factors associated with SA in adolescence. We formulated the following hypotheses: (i) that boys would show more externalized disorders while girls would show more internalized disorders; (ii) that boys would use more non-productive coping strategies while girls would make more use of Reference to Others strategies. We did not formulate a hypothesis for spirituality.

## Methods

### Participants

Our study sample was made up of adolescents aged 13–17 admitted to a pediatric department following a suicide attempt and included in a longitudinal multisite study designed to assess the risk and protective factors associated with suicidal recidivism from January 2011 to December 2014 [detailed protocol in Mirkovic et al. (29)]. According to the French recommendations of good practice [issued by the HAS–Haute Autorité de Santé 1998 (40)], all adolescents who visit an emergency department for suicidal behavior are hospitalized. We considered the event as a SA only if intent to die was manifest. Subjects committing non-suicidal self-injury without intent to die were excluded. The adolescents also had to be able to understand French, live in the geographic area of the recruiting center, have no mental retardation or neurological disorder and have given their written consent. To participate in the study, under-age subjects needed to have the agreement of both parents or guardians. The exclusion criteria included an inability to provide written informed consent (for example, moderate to severe cognitive impairment) or a current medical condition or residence outside of the geographical area of each center.

The five pediatric departments involved belonged to two university hospitals and three peripheral hospitals. All five hospitals cover both urban and rural geographical areas in three French regions.

### Procedures

During hospitalization, participation in the study was proposed systematically, by their referent psychiatrist, to all adolescents who met the inclusion criteria. After obtaining the consent of the adolescent and both parents, the research team contacted the patient and his/her family. The evaluation was conducted during hospitalization by psychiatrists trained in conducting semi-structured interviews and questionnaires as defined in the study protocol. To avoid tiring the patients, the evaluation was carried out over more than one interview session. The investigators were senior psychiatrists and psychiatry residents. All the evaluation reports were analyzed individually by our study group.

### Measurements

#### Protective Factors

*The Adolescent Coping Scale* (ACS) was used to assess specific behaviors adopted to deal with stressful life events. The ACS was designed and validated for adolescents aged 12–18 (32). The ACS assesses the specific behaviors used to cope with a situation or to solve a problem. According to the instrument manual, the three coping styles (productive coping, non-productive coping and reference to others) show sufficient internal coherence to justify their separate subscales (with alphas ranging from 0.62 to 0.75). The ACS has also demonstrated its reliability among 146 suicidal adolescents (31).

*The Spirituality Scale* (41) was used to score spiritual beliefs, self-discovery, self-awareness and collective consciousness, and respect for others and environment. The construct of spirituality proposed by Delaney (41) is broad. It goes beyond religious practices and encompasses three key relational aspects: connection with self (personal), with others (interpersonal), and with the divine (transpersonal). Possible scoring on the 23-item Spirituality scale ranged from 23 to 138. The scores indicated how important the phenomenon of spirituality is to, or is manifested by, the person. Scores between 23 and 60 indicated very low levels of spirituality, 61–91 indicated low spirituality, 92–117 indicated moderate spirituality and 118–138 suggested high levels of spirituality or spiritual wellness. Labelle et al. (31) have shown that this instrument is appropriate for assessing protective factors against depression and suicidal behavior in French-speaking adolescents in community (*n* = 283) and clinical settings (*n* = 146).

#### Risk Factors for SB

Axis I psychiatric disorders were evaluated with *the Scale of Mood Disorders and Schizophrenia for Children and Adolescents of School Age, Current and Past Episodes* version (Kiddie-SADS-PL) (42). Depressive and hopelessness severity were measured, respectively, with the *Beck Depression Inventory* (BDI) second edition (43) and the Beck Hopelessness Scale (BHS) (44).

Borderline personality disorders were assessed by *Abbreviated-DIB (Ab-DIB)* (45). It is a 10-min Diagnosis Interview for Borderline-Revised (DIB-R) derived 26-item self-report inventory used to assess impulsiveness, affective and cognitive components of borderline personality symptomatology.

Suicide characteristics were based on: *the clinician-administered Columbia-Suicidal Severity Rating Scale (C-SSRS*) (46) to assess the severity of suicidal ideations in the past month, actual attempt behavior severity, the number and characteristics of past attempts and Non-Suicidal Self Injury.

Impulsivity was assessed using the impulsivity section of the *Eysenck Questionnaire* (47). This 24-item self-administered questionnaire, completed by 8–17-year-olds, makes use of the norms established in two studies in the general Canadian and British population.

*The Life Events Questionnaire* (48) is a 39-item instrument used to assess recent stressful situations experienced by adolescents (14–18 years-olds).

Self-esteem was assessed with the *Rosenberg Self-Esteem Rating Questionnaire* (49) and attachment style with the *Relationship Scales Questionnaire* (RSQ) (50).

### Data-Analytic Strategy

Descriptive statistics of sample characteristics, SA methods and risk and protective factors for SA were analyzed. We compared sociodemographic and clinical differences between boys and girls, using an independent *t*-test, Wilcoxon Rank-Sum test or ANOVA for continuous variables and the chi-square or Fisher's exact tests for categorical variables. In statistical analyses, a significance level of 0.05 was applied. We modeled the number of suicidal attempts using a Poisson regression (Model formula was: SA-number ~ Total_BDI + Gender + Total_spirituality + Gender^*^Total_spirituality). The statistical package SPSS Release 16.0.2 (SPSS Inc., Chicago, IL, USA; 2008) and R 3.4.0 were used for the entire analyses.

## Results

Participation in the study was proposed to 398 eligible adolescents between February 2011 and December 2015. Three hundred twenty subjects were included, with 265 girls (82%) and 55 boys (18%), and the mean age was 14.43 (SD 1.29).

The 78 eligible adolescents who did not participate in the study made this choice for the following reasons: adolescent refused (*n* = 41), parent refused (*n* = 20), consent withdrawn during hospitalization (*n* = 8), others (*n* = 9). The sociodemographics of those who declined participation were roughly comparable to those of the participants regarding mean age (participants = 14.6 years vs. non-participants = 15.1 years) and gender (girl participants = 82% vs. girl non-participants = 79%).

The number of adolescents by center was as follows: CHU Rouen: 177 (55%); CHU Amiens: 80 (25%); CH Compiègne: 32 (10%); CH Creil: 22 (7%); CH Maux: 9 (3%). Of the 320 subjects included, 112 (35%) had already made a first SA. Table 1 summarizes the main sociodemographic characteristics of the entire sample and comparisons made by gender. We found only one sociodemographic variable which differed according to gender. Boys have greater difficulties at school or more precisely, they have more often repeated a school year χ2 (319) = 5.03, (*p* = 0.025). Table 2 give the principal SA characteristics on the basis of the Columbia Suicide Severity Rating Scale. Boys more often resort to strangulation and girls to laceration. Girls are significantly younger at their first suicide attempt (*p* = 0.048) and they make more repeat attempts (*p* = 0.024).

**Table 1:** Socio-demographic characteristics and comparisons by gender.

**Characteristics**	**Total**	**Girls**	**Boys**	***p***
	**(*N* = 320)**	**(*N* = 265)**	**(*N* = 55)**	
Mean age (SD)	14.43 (1.29)	14.65 (1.89)	14.14 (1.52)	ns
Number of children in family (SD)	2.46 (1.27)	2.44 (1.3)	2.44 (1.3)	ns
School-year repeaters *N* (%)	101 (31.5)	77 (29.06)	24 (43.64)	0.025
Residence with both parents *N* (%)	132 (41.2)	107 (40.38)	25 (45.45)	ns
Normal school career *N* (%)	271 (84.6)	228 (86.04)	43 (78.18)	ns
Mother's level of education *N* (%)^*^				
1	32 (10)	29 (10.94)	3 (5.45)	ns
2	88 (27.5)	74 (27.92)	14 (25.45)	
3	94 (29.3)	78 (29.43)	16 (29.09)	
4	65 (20.3)	52 (19.62)	13 (23.64)	
5	41 (12.8)	32 (12.08)	9 (16.36)	
Father's level of education *N* (%)^*^				
1	39 (12.1)	33 (12.45)	6 (10.91)	ns
2	96 (30)	81 (30.57)	15 (27.27)	
3	74 (23.1)	59 (22.26)	15 (27.27)	
4	63 (16.7)	53 (20)	10 (18.18)	
5	48 (15)	39 (14.72)	9 (16.36)	
Had previous psychiatric care *N* (%)	100 (31.2)	82 (30.94)	18 (32.73)	ns

* *1, primary and lower secondary school; 2, higher secondary school; 3, secondary professional school; 4, tertiary education (around 2–3 years more after high school diploma); 5, tertiary education (around 5–6 years more after high school diploma)*.

**Table 2:** Characteristics of suicidal behaviors evaluated on the Columbia-suicide severity rating scale and comparisons by gender.

**Characteristics**	**Total**	**Girls**	**Boys**	***p***
	**(*N* = 320)**	**(*N* = 265)**	**(*N* = 55)**	
Ideations *N* (%)				
None	89 (27.8)	73 (27.55)	16 (29.0)	ns
Passive^*^	89 (27.8)	75 (28.3)	14 (25.6)	
Active^**^	142 (14.4)	117 (44.15)	25 (45.4)	
Intentionality *N* (%)				
Hidden	53 (16.56)	63 (23.77)	12 (21.82)	ns
Ambivalent	149 (46.5)	139 (52.45)	22 (40)	
Definitive	107 (33.4)	63 (23.77)	21 (38.18)	
Thoughts *N* (%)				
None	181 (56.5)	151 (56.98)	30 (54.55)	ns
Phobia	57 (17.8)	52 (19.62)	5 (9.09)	
Suicide-related	82 (25.6)	62 (23.4)	20 (36.36)	
Method *N* (%)				
Electrocution	5 (1.5)	4 (1.51)	1 (1.82)	0.007
Poisoning	242 (75.6)	205 (77.36)	37 (67.27)	
Laceration	20 (6.2)	18 (6.79)	2 (3.64)	
Jumping	13 (4)	13 (4.91)	0 (0)	
Strangulation	28 (8.7)	17 (6.42)	11 (20)	
Other	12 (3.7)	8 (3.02)	4 (7.27)	
Number of SAs *N* (%)				
1	208 (65)	170 (64.15)	38 (69.09)	0.024
2	59 (18.6)	52 (19.62)	7 (12.73)	
3	26 (8.1)	25 (9.43)	1 (1.82)	
4	13 (4)	8 (3.02)	5 (9.09)	
5	8 (2.5)	7 (2.64)	1 (1.82)	
6	3 (0.9)	1 (0.38)	2 (3.64)	
7	3 (0.9)	2 (0.75)	1 (1.82)	
Mean number of SAs *N* (%)	1.67 (1.18)	1.65 (1.11)	1.8 (1.51)	ns
Mean age at first SA (SD)	14.48 (1.42)	14.65 (1.45)	15.07 (1.39)	0.048

* *implies a desire to die, but without a specific plan to carry out death*.

** *implies an existing desire to die accompanied by a plan for how to carry out death*.

*SA, Suicide Attempt*.

### Differences in Coping Styles and Strategies

We performed the Wilcoxon Rank-Sum test to explore differences in coping styles and strategies between girls and boys. Girls reported more frequent use of the *Reference to others* coping style than boys did W(302) = 7541.5, (*p* = 0.045). Of the strategies of the *Reference to others* style, only *seeking social support* was significant W(302) = 7692.5, (*p* = 0.023). As regards unproductive coping, we identified two significant differences between the two groups: *wishful thinking* W(302) = 5,013 (*p* = 0.049) and *tension reduction* W(317) = 7,553, (*p* = 0.042).

### Differences in Spirituality

Both groups had low spirituality scores (<91). Girls had a significantly higher overall spirituality score than boys W(317) = 7,208, *p* = 0.027. The results indicate that girls reported more *self-awareness and collective consciousness* W(317) = 7,263, *p* = 0.02 and *respect of others and environment* W(317) = 7434.5, *p* = 0.008 than boys did.

### Axis I Diagnoses and Borderline Personality Disorders

For the two groups, we compared all the diagnoses assessed with the Kiddie-SADS-PL. We found no significant difference between the two groups (Table 3). As regards borderline personality disorder assessed with the Ab-DIB, girls fitted the diagnosis significantly better than boys, respectively, *n* = 191 (73.7%) and *n* = 32 (60.4%), χ2 (312) = 3.86, *p* = 0.05.

**Table 3:** Individual risk factors and comparisons by gender.

**Characteristics**	**Total**	**Girls**	**Boys**	***p***
	**(*N* = 320)**	**(*N* = 265)**	**(*N* = 55)**	
Axis I diagnoses (DSM-IV-R) Yes, *N* (%)				
Major depressive disorders	131 (41%)	105(41.5%)	25(48.1%)	ns
Anxiety disorders	88 (28%)	70(27.7%)	12(22.6%)	ns
Psychotic disorders	3 (1%)	3(1.2%)	2(3.8%)	ns
Substance and alcohol-related disorders	41 (13%)	72(28.5%)	20(38.5%)	ns
Disruptive and oppositioal behavior disorder	66 (21%)	55(21.7%)	8(15.1%)	ns
Attention deficit hyperactivity disorder	17 (5%)	12(4.7%)	4(7.5%)	ns
Borderline disorder (Ad-DIB) *N* (%)				
No	89 (28%)	68 (26.3%)	21 (39.6%)	0.05
Yes	224 (70%)	191 (73.7%)	32 (60.4%)	
Dimensional assessments				
Beck Depression Inventory-II, Mean (SD)	25.3 (13.9)	25.7 (13.6)	23.15 (14.1)	ns
Beck Hopelessness Scale, Mean (SD)	9.35 (5.5)	9.28 (5.55)	9.62 (4.77)	ns
Life Events Questionnaire for Adolescents, Mean (SD)	3.5 (2.3)	3.62 (2.22)	3.27 (1.89)	ns
Dependance, Mean (SD)	7.3 (7.2)	7.38 (6.93)	6.49 (6.1)	ns
Impulsivity, Mean (SD)	12.2 (4.75)	12.34 (4.75)	11.58 (4.83)	ns
Secure attachment, Mean (SD)	13.4 (3.11)	13.7 (3.49)	13.4 (3.26)	ns
Fearful attachment, Mean (SD)	11.6 (3.42)	11.78 (3.72)	11.55 (3.44)	ns
Dimissing attachment, Mean (SD)	12.05 (3.08)	12.11 (3.28)	11.81 (3.21)	ns
Preoccupied attachment, Mean (SD)	16.4 (3.5)	16.84 (3.94)	16.32 (3.5)	ns
Self estime, Mean (SD)	5.95 (2.3)	6.21(2.41)	4.93(2.2)	ns

### Dimensional Characteristics

We found no significant differences between boys and girls for: the Beck Depression Inventory-II, the Beck Hopelessness Scale, the Life Events Questionnaire for Adolescents, and the dependence, impulsivity, self-esteem and attachment style (Table 3).

### Regression Models

We conducted linear regressions to see how spirituality influences the number of suicides attempts according to gender and adjusting for depression (Beck Depression inventory). On average, Spirituality (total score) effect on the mean number of suicidal attempts is significantly increased for males (vs. females) by a factor 1.023, *p* = 0.002 (Estimate = 0.023, Std. Error = 0.007, *z*-value = 3.151). Spirituality effect on the mean number of suicidal attempts in females is not significant, *p* = 0.810 (Estimate = −0.001, Std. Error = 0.003, *z*-value = −0.241). An increase of one point of total spirituality in males increases the mean number of suicidal attempts by a factor 1.022, *p* = 0.001, (Estimate = 0.022, Std. Error = 0.007, *z*-value = 3.393) see Table 4.

**Table 4:** Individual protective factors and comparisons by gender (Wilcoxon Rank-Sum test).

	**Total**		**Girls**		**Boys**		
	**(*****N*** **= 320)**		**(*****N*** **= 265)**		**(*****N*** **= 55)**		***p***
	**Mean**	**SD**	**Mean**	**SD**	**Mean**	**SD**	
**Spirituality scale**				
Spiritual beliefs	17.84	8.09	18.08	8.01	16.73	8.44	ns
Self-discovery	18.97	4.78	19.06	4.72	18.51	5.12	ns
Self-awareness and	14.52	5.03	14.87	4.89	12.9	5.42	0.008
collective consciousness							
Respect of others and	18.61	3.87	18.83	3.79	16.59	4.1	0.02
environment							
Total	69.97	16.63	70.85	16.27	65.73	17.78	0.027
**Adolescent coping scale**				
Productive coping (total)	59.35	13.93	59.44	13.75	58.71	14.97	ns
Focus on solving problem	51.12	16.06	51.41	15.85	49.1	16.82	ns
Work hard and achieve	62.35	17.41	62.85	17.07	59.92	19.14	ns
Focus on the positive	50.80	17.63	51.01	17.55	49.4	18.06	ns
Seek relaxing diversions	73.45	19.66	73.23	19.56	74.34	20.44	ns
Physical recreation	60.62	24.17	60.27	23.19	62.59	28.84	ns
*Non*-productive coping	54.42	12.10	54.65	12.27	53.22	11.39	ns
(total)							
Worry	52.66	17.42	52.7	17.9	52.63	17.13	ns
Seek to belong	57.03	15.35	57.42	15.31	54.64	15.17	ns
Wishful thinking	49.95	17.45	48.25	17.44	53.76	17.05	0.049
Not coping	50.07	16.69	50.8	17.22	46.27	14.84	ns
Tension reduction	52.50	17.28	53.42	17.56	48.08	15.46	0.042
Ignore the problem	49.10	17.13	48.66	17.03	51.1	17.7	ns
Self-blame	60.46	19.72	60.98	19.9	57.65	18.8	ns
Keep to self	67.38	20.60	67.91	20.61	64.22	20.31	ns
Reference to others (total)	41.35	10.82	42.19	10.49	40.23	12.36	0.045
Invest in close friends	58.51	17.61	59.25	17.5	56.94	19.95	ns
Seek social support	49.9	18.42	51	18.5	44.63	17.37	0.023
Seek spiritual support	30.33	17.71	30.82	18.25	28.14	14.86	ns
Social action	30.92	11.23	30.64	10.64	32.2	13.89	ns
Seek professional help	39.66	18.99	39.59	19.04	39.7	18.96	ns

## Discussion

In this study we examined gender difference for the risk and protective factors associated with adolescent SAs. We included 320 adolescents, 82% of whom were girls, during hospitalization for AS.

Regarding protective factors, we showed that girls make more use of the Reference to Others coping style, mainly the *seeking social support* strategy, while men make more use of the non-productive coping style, mainly *wishful thinking* and *tension reduction* strategies. Lastly, regarding spirituality, although the scores were generally low in both groups, the girls had significantly higher scores, especially for questions related to *self-awareness* and *collective consciousness*.

Our epidemiological data agree with the data from the literature. Our sample is composed mainly of girls (82%) and 35% of repeat attempters (3, 9, 51). Although the repeat attempter rate is higher than that reported by other teams, it is consistent with other studies including high-risk adolescents, such as King et al. (51). Regarding risk factors, we found very few differences by gender. We failed to find any difference for the main psychiatric diagnoses between the two groups. This result does not support recent studies which have shown a predominance of internalized disorders in women (23). Our divergent results may be explained in part by the nature of our sample. Indeed, our cohort consists only of adolescents hospitalized for attempted suicide. It is therefore a severe clinical sample with a high prevalence of depressive disorders and severe overall functioning. In this context of severity, gender differences are more difficult to demonstrate compared to community samples. Furthermore, this result is in agreement with the recent study by Miranda-Mendizabal et al. (52) which showed that mood disorders are the only risk factors for suicidal thoughts that are common to both sexes.

We found differences in SA patterns that have been widely reported by other teams (4, 5, 25). Overall, boys use more lethal methods such as strangulation while girls use mostly drug poisoning.

As regards coping skills, we showed that girls are more likely to seek help from their entourage (*seeking social support)* than boys. Gould et al. (53), in a self-report survey on the self-management of suicidal behavior in the general population (among secondary school students), showed that girls had better results for help-seeking strategies. In addition, men and women show differences in what they perceive as barriers to seeking help. For example, stigma and embarrassment are more dissuasive for men than for women (54). This is particularly important because several studies have shown the protective role of the perception of social or family support in the face of suicidal behavior (55, 56). Yet support can only be provided if there is a prior request for help.

Finally, concerning spirituality, our results contribute to furthering the relatively limited knowledge of gender differences. Girls had higher scores, especially for *self-awareness, collective awareness* and *respect of others and environment*.

In our regression model, spirituality can be considered a risk factor for suicidality only for boys. For girls there was no significant effect. Our results are not in agreement with other similar population-based studies that have shown spirituality to be a protective factor (27, 39). However, in the study by Breton et al. (39) only the subscale *Self-discovery* was a significant protective factor for suicide attempt. In adolescents, spirituality seems to have a versatile effect on suicidality and this effect is different for girls and boys. The construct of spirituality proposed by Delaney encompasses three key relational aspects: connection with self (personal), with others (interpersonal), and with he divine (transpersonal) and goes far beyond religious practices. In contrast to spirituality, the protective effect of religion against suicidal risk has been reported in many studies (57–59). Among the underlying reasons for this effect, various factors have been suggested: religious beliefs, church attendance and participation in public religious practices, moral objections to suicide, lower levels of aggressiveness, and fewer addictions.

The study by Kralovec et al. (57), the only one to have studied gender-specific associations between religion/spirituality and suicide risk in a clinical sample, showed that religion/spirituality was correlated protectively with suicide risk, with stronger associations among women. More precisely, the authors found that most dimensions of religiousness were inversely correlated with many of the factors described in suicide models. This indicates that women's risk of suicide may not be primarily affected by church attendance, but by a wide range of religious dimensions, including belief/ideology, religious experience and prayer.

Previous studies have reported protective effects of other religious dimensions in addition to church attendance. In adolescents, private religiousness (prayer and the importance of religion) was significantly associated with fewer suicidal thoughts and fewer suicide attempts (58). Moreover, Caribe et al. (59) reported fewer suicide attempts among those engaged in both non-organized religious activities, in particular prayer or meditation, and religious activities within an organization.

The large number of participants constitutes a strength of the present study. However, it has some limitations. The major limitation of the study is the fact that it was a cross-sectional study. This is important, as no causal claims can be inferred from the statistical associations identified in the study. Moreover, adolescents hospitalized for attempted suicide may differ from those who are not (Berkson's selection bias). Added to that, the self-assessment methodology used may be subject to a registration bias, such as social desirability.

## Conclusions

Our results highlight differences between boys and girls as regards spirituality and certain coping strategies. Specifically, girls are more likely to use *seeking social support* strategies than boys. Boys are more likely to use unproductive coping strategies such as *tension reduction* (substance abuse) and *wishful thinking* (avoidance strategies. Individuation of interventions represents a promising strategy to counter the misuse of gender roles to tailor suicide prevention programs. School mental health classes can be adapted to consider different approaches for males and females adolescent, for example, by valuing help-seeking strategies for young males. By ensuring the personalization of suicide prevention programs, mental health professionals can expect greater responsiveness and, in theory, a decrease in suicide attempts and suicides among youth.

## Data Availability Statement

The datasets generated for this study are available on request to the corresponding author.

## Ethics Statement

Ethics approval was obtained for the study from the North West I (Charles Nicolle CHU -University Hospital) Group Ethics and Medical Research Committee (2010 A00 330 - 39). Written informed consent to participate in this study was provided by the participants' legal guardian/next of kin.

## Author Contributions

BM and VB participated in the conception of the manuscript, coordinated the data collection, conducted the data analyses, provided drafts of the manuscript, and finalized the manuscript. J-MG participated in the design and coordination of the study and performed the measurements. HP performed the statistical analysis. PG conceived of the study, participated in its design and coordination and helped to draft the manuscript. All authors read and approved the final manuscript.

## Conflict of Interest

The authors declare that the research was conducted in the absence of any commercial or financial relationships that could be construed as a potential conflict of interest.
